# Improved predictions of total kidney volume growth rate in ADPKD using two-parameter least squares fitting

**DOI:** 10.1038/s41598-024-62776-8

**Published:** 2024-06-14

**Authors:** Zhongxiu Hu, Arman Sharbatdaran, Xinzi He, Chenglin Zhu, Jon D. Blumenfeld, Hanna Rennert, Zhengmao Zhang, Andrew Ramnauth, Daniil Shimonov, James M. Chevalier, Martin R. Prince

**Affiliations:** 1 Department of Radiology, Weill Cornell Medicine, New York, 10022 USA; 2 The Rogosin Institute, New York, 10021 USA; 3 Department of Medicine, Weill Cornell Medicine, New York, 10021 USA; 4 Department of Pathology and Laboratory Medicine, Weill Cornell Medicine, New York, 10065 USA; 5 Department of Radiology, Columbia University Vagelos College of Physicians and Surgeons, New York, 10032 USA

**Keywords:** Nephrology, Chronic kidney disease, Polycystic kidney disease, Predictive markers, Prognostic markers

## Abstract

Mayo Imaging Classification (MIC) for predicting future kidney growth in autosomal dominant polycystic kidney disease (ADPKD) patients is calculated from a single MRI/CT scan assuming exponential kidney volume growth and height-adjusted total kidney volume at birth to be 150 mL/m. However, when multiple scans are available, how this information should be combined to improve prediction accuracy is unclear. Herein, we studied ADPKD subjects ($n = 36$) with 8+ years imaging follow-up (mean = 11 years) to establish ground truth kidney growth trajectory. MIC annual kidney growth rate predictions were compared to ground truth as well as 1- and 2-parameter least squares fitting. The annualized mean absolute error in MIC for predicting total kidney volume growth rate was $2.1\% \pm 2\%$ compared to $1.1\% \pm 1\%$ ($p = 0.002$) for a 2-parameter fit to the same exponential growth curve used for MIC when 4 measurements were available or $1.4\% \pm 1\%$ ($p = 0.01$) with 3 measurements averaging together with MIC. On univariate analysis, male sex ($p = 0.05$) and *PKD2* mutation ($p = 0.04$) were associated with poorer MIC performance. In ADPKD patients with 3 or more CT/MRI scans, 2-parameter least squares fitting predicted kidney volume growth rate better than MIC, especially in males and with *PKD2* mutations where MIC was less accurate.

## Introduction

Autosomal dominant polycystic disease (ADPKD), the most prevalent kidney genetic disorder, affects 12 million people worldwide and accounts for 5-10% of all cases of end stage kidney disease (ESKD)^1^. Disease progression in typical ADPKD is routinely estimated by Mayo Imaging Classification (MIC) based on a single CT or MRI of the abdomen together with patient age and height^2–5^. MIC assumes that height-adjusted total kidney volume (htTKV) follows an exponential trajectory starting at a htTKV at birth of 150 mL/m with classes defined as 1A (htTKV growth rate (*r*) less than $< 1.5\%$ per year), 1B ($1.5\% \le r < 3\%$ per year), 1C ($3\% \le r < 4.5\%$ per year), 1D ($4.5\% \le r < 6\%$ per year) and 1E ($r \ge 6\%$ per year)^6^. Some authors recommend using MIC to decide which patients are rapid progressors (i.e., classes 1C-1E) and thus suitable for treatment with tolvaptan^7,8^.

Since uncertainty in htTKV measurement affects accuracy when calculating the rate of increase in htTKV, it is essential to have precise and reproducible htTKV measurements. Deep learning methods have emerged as the most reproducible approach to measuring htTKV, exceeding even manual segmentation by the radiologist^9–18^. Nevertheless, there can still be MIC variability from scan to scan. Irazabal et al. found that MIC changed in 10% of ADPKD subjects after a median follow-up of 4 years^6^. Other studies also reported scan to scan variability in MIC class^19,20^.

In this study, we identified ADPKD subjects with over 8 years of cross-sectional imaging follow-up to establish a ground truth annual htTKV growth rate. This ground truth was then compared to MIC using the earliest available scan, MIC using the latest scan, averaging MIC and least squares fitting.

## Results

**Figure 1:**
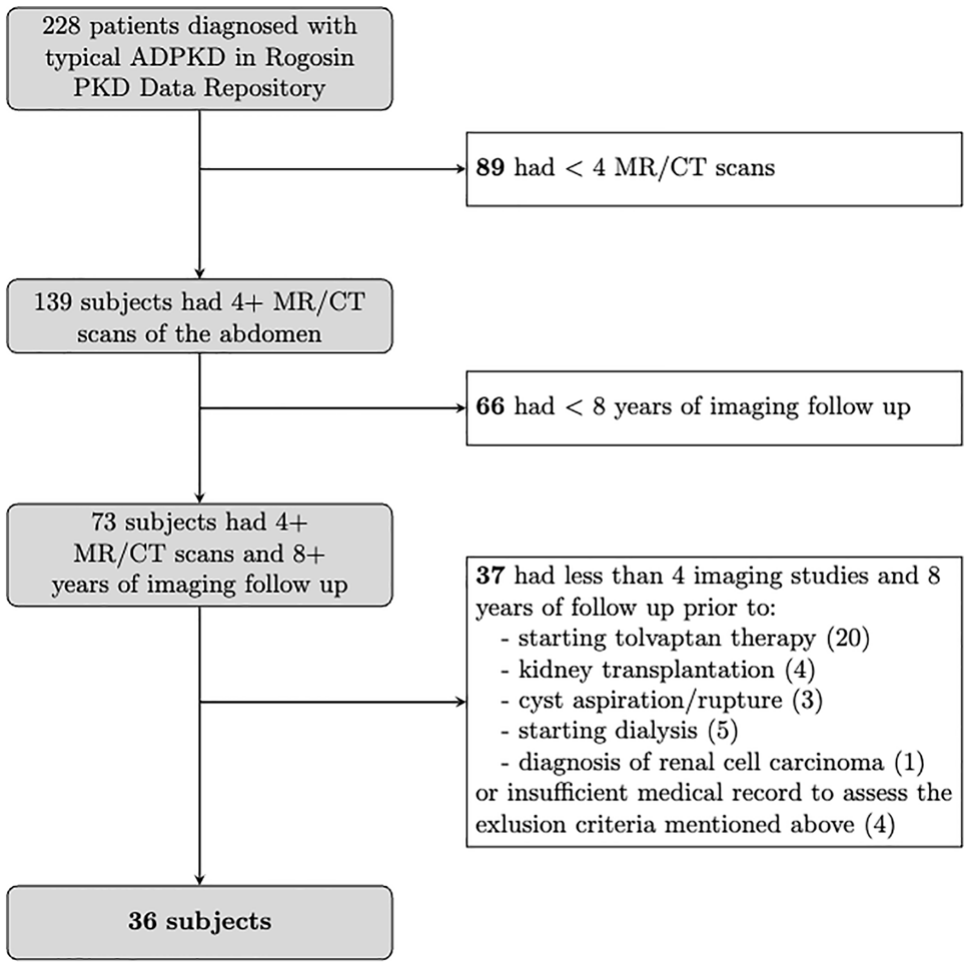
Flow chart of the study population selection.

Thirty-six subjects (Fig. 1) fulfilled the inclusion and exclusion criteria. Demographic data are shown in Table 1 for all 36 subjects grouped according to how well MIC calculated from the earliest scan available (index scan) aligned with ground truth (Supplemental Fig. S1).

**Table 1:** Subject Demographics: median values and [interquartile range] for all subjects and groups based on absolute difference in htTKV growth rate from Mayo Imaging Classification (MIC) prediction of the first available scan to ground truth.

	All ($n = 36$)	Ideal MIC alignment, < 0.75% ($n = 7$)	Slight MIC misalignment ($n = 7$)	Substantial MIC misalignment, > 1.5% ($n = 22$)
htTKV growth rate (% per year)	3.6 [2.8 5.3]	2.0 [1.5 4.2]	3.4 [2.8 4.5]	4.1 [3.2 6.2]
Age at index scan	41 [32 53]	32 [30 50]	41 [31 50]	43 [37 57]
Age at most recent scan	53 [44 64]	47 [51 56]	46 [39 47]	60 [59 71]
Male : Female (%Male)	21:15 (58%)	1:6 (14%)	5:2 (71%)	15:7* (68%)
Years of follow-up	11 [10 13]	14 [10 16]	10 [8 10]	11 [10 13]
# of scans	6.0 [5.0 8.0]	7.0 [6.5 8.0]	5.0 [4.0 6.0]	6.0 [5.0 8.0]
Progression Faster; Slower than MIC	24; 5 (65%;14%)	−	6; 1 (16%; 3%)	18; 4 (49%; 11%)
htTKV at index scan (mL)	392 [312 738]	359 [226 832]	384 [333 576]	405 [330 576]
htTKV at most recent scan (mL)	635 [447 1393]	475 [268 1524]	633 [484 746]	934 [495 1499]
eGFR at 1st scan (mL/min/1.73 m^2^)	93 [68 110]	83 [73 108]	109 [92 118]	93 [55 108]
eGFR at last scan (mL/min/1.73 m^2^)	71 [36 90]	48 [27 78]	90 [64 98]	70 [33 83]
Urine osmolality at last scan (mOsm/kg H_2_O)	325 [253 541]	283 [264 316]	493 [290 687]	365 [166 539]
Hypertension diagnosis	29 (81%)	4 (57%)	6 (86%)	19 (86%)
# of subjects with exophytic cysts	30 (86%)	6 (88%)	5 (71%)	19 (86%)
# of T1 bright cysts at index scan	3 [0 12]	6 [1 17]	1 [0 10]	3 [0 12]
Genotype				
*PKD1* truncating	5	2	1	2
*PKD1* non-truncating	10	2	4	4
*PKD2* truncating	7	0	0	7
*PKD2* non-truncating	4	1	0	3
Other^a^	10	2	2	6
Mayo Imaging Class on initial MRI/CT scan				
1A	10	3	2	5
1B	10	1	1	8
1C	9	2	3	4
1D	3	0	1	2
1E	4	1	0	3

Ideal alignment means the annual growth rate predicted by the MIC calculation varies less than 0.75% per year from ground truth, i.e. variation within 1 MIC band; Slight MIC misalignment varies $\ge$ 0.75% but less than 1.5% (within 2 MIC bands) and substantial MIC misalignment varies $\ge$ 1.5% from ground truth. Benjamini-Hochberg method was used to adjust *p*-values to account for multiplicity of statistical tests.

^a^The genetic result was inconclusive or not performed.

*Statistically significantly different from ideal MIC alignment with $p < 0.05$.

### Characteristics of patients of ideal alignment, slight or substantial misalignment of MIC

Substantial misalignment of MIC from ground truth, $n = 22$ (60%) with $> 1.5\%$ absolute error in the annual rate of htTKV increase, was significantly more common in male ADPKD subjects ($p = 0.05$) and there was a trend toward older age at first imaging study ($p = 0.16$). Patients with *PKD2* mutation were also more likely to have substantial misalignment of MIC from ground truth ($p = 0.04$, Table 1). Notably, the only *PKD2* mutation case with ideal alignment with MIC was non-truncating (Table 1). The majority (75%) of *PKD1* patients progressed with ideal alignment ($n = 4$) or only slight misalignment ($n = 5$) from MIC, yet 90% (9 of 10) of *PKD2* patients were substantially misaligned.

Although only spot urine osmolality measurements at the time of the most recent scans were available, there was a trend toward higher urine osmolality for patients substantially deviating from MIC predictions with faster htTKV growth rate than predicted by MIC (on average 404 mOsm/kg H_2_O, $n = 18$) compared to a mean of 289 mOsm/kg H_2_O ($p = 0.16$) in patients with ideal alignment to MIC. Average urine osmolality in males was 402 mOsm/kg H_2_O compared to 366 mOsm/kg H_2_O in females (*p* = 0.33). There was also a trend for ADPKD patients diagnosed with hypertension to substantially deviate from MIC predictions ($p = 0.16$, Table 1).

We found no relationship between exophytic cysts, complex (T1 bright) cysts, eGFR, htTKV and the degree of alignment to MIC.

### Accuracy of estimated htTKV growth rate

The ground truth annual htTKV growth rate for the 36 ADPKD subjects ranged from -0.5% to 11.6%, median = 3.6%. Table 2 shows absolute error in htTKV annual growth rate calculated by each of the 4 methods for every timepoint after the first scan averaging all assessments, (first line, $n=198$) as well as organized by genotype, sex, number of years of imaging follow-up and by the number of imaging timepoints being used to calculate annual TKV growth rate. Averaging MIC, Method 2, and the 1-parameter and 2-parameter fitting, Methods 3 and 4, required at least 2 scans (timepoints) to calculate annual htTKV growth rate so they could not be calculated for the first scan. These data show the 2-parameter least squares fit had the least absolute error overall (0.98% ± 1.6%) which was less than half the mean error of the other three methods. However, because the 2-parameter fit performs better with more data, when there were only 2 timepoints or less 5 years of follow-up imaging, the 2-parameter fit performed worst and MIC was the best choice.

Absolute error in the calculation of annual htTKV growth rate was greater for *PKD2* mutations compared to *PKD1* mutations for MIC, averaging MIC, and the 1-parameter least squares fit. The performance of the 2-parameter fit on *PKD1* and *PKD2* was similar. All methods showed greater error in the males compared to females except in the 2-parameter fit which performed similarly in males compared to females.

For Method 1 (MIC), 15 out of 36 patients (42%) eventually had a different MIC compared to the MIC calculated from their first scan: MICs of 7 subjects changed after acquiring the second imaging study, and MICs of an additional 3, 2, and 2 subjects changed after the third, fourth, and fifth scans respectively.

There was no reduction in error from averaging MICs of each available timepoint (absolute error $2.3\% \pm 2\%$ per year) compared to calculating htTKV growth rate using the htTKV measured from the latest scan (absolute error $2.1\% \pm 2\%$ per year). There was a trend toward improved htTKV growth rate estimation by MIC using the most recent scan compared to using the index scan (absolute error $1.8\%\pm 1\%$ per year, $p = 0.10$).

Single parameter least squares fitting attained no improvement over MIC. However, the 2-parameter least squares fitting with 4 timepoints available (absolute error $1.1\% \pm 2\%$ per year) had superior accuracy compared to the average absolute error of using MIC on the latest scan ($2.0\% \pm 2\%$ per year, $p=0.002$), improving further with additional available timepoints. Superior accuracy with 2-parameter fitting was also attained with 5-7 years or more imaging follow-up.

**Table 2:** Absolute error (± standard deviation) in calculated annual height adjusted total kidney volume (htTKV) growth rate using 1. Mayo Imaging Classification (MIC) of the latest timepoint, 2. averaging htTKV growth rates calculated using MIC, 3. 1-parameter least squares fitting (1-param. LSF) according to the MIC assumption that htTKV_0_ = 150 mL/m, 4. 2-parameter least squares fitting (2-param. LSF) evaluated using 2 to *n* timepoints and measured htTKV available, and averaging the rate of htTKV increase calculated by Method 1 and 4.

	*n*	Absolute Error in htTKV annual growth rate (% per year)				
		1. MIC latest timepoint	2. Avg. MIC	3. 1-param. LSF	4. 2-param. LSF	Avg. 1 &4
All Assessments	198	2.09 ± 1.7	2.26 ± 1.8	2.25 ± 1.8	0.98 ± 1.6***†	–
a. Genotype						
*PKD1*	89	1.87 ± 1.4	2.01 ± 1.6	2.00 ± 1.5	1.01 ± 1.3***†	–
*PKD2*	54	2.44 ± 1.5	2.62 ± 1.6	2.61 ± 1.6	0.78 ± 1.3***†	–
Other	55	2.08 ± 2.1	2.28 ± 2.3	2.26 ± 2.3	1.14 ± 1.7*†	–
b. Sex						
Male	104	2.50 ± 1.8	2.70 ± 2.0	2.69 ± 2.0	0.83 ± 1.5***†	–
Female	94	1.64 ± 1.3	1.76 ± 1.5	1.75 ± 1.5	1.14 ± 1.7*†	–
c. Years of imaging						
< 3 years	21	2.57 ± 2.0	2.64 ± 2.1	2.64 ± 2.1	7.91 ± 10.3	3.72 ± 5.6
3–5 years	50	2.19 ± 1.4	2.27 ± 1.6	2.26 ± 1.6	2.41 ± 2.1	1.77 ± 1.4
5–7 years	33	2.38 ± 2.0	2.52 ± 2.1	2.51 ± 2.1	1.09 ± 1.0**	1.49 ± 1.1
7–9 years	28	1.61 ± 1.2	1.79 ± 1.4	1.78 ± 1.4	0.49 ± 0.5***	0.90 ± 0.5
9–12 years	42	1.83 ± 1.2	2.06 ± 1.4	2.04 ± 1.4	0.26 ± 0.4***	0.93 ± 0.6
12–15 years	14	2.43 ± 2.4	2.86 ± 2.8	2.82 ± 2.7	0.21 ± 0.2**	1.18 ± 1.2
15+ years	10	1.59 ± 1.9	1.81 ± 2.2	1.79 ± 2.2	0.03 ± 0.1*	0.80 ± 0.9
d. # of Imaging timepoints						
1 timepoint	36	2.31 ± 2.0	–	–	–	–
2 timepoints	36	2.29 ± 1.8	2.33 ± 1.9	2.32 ± 1.9	5.35 ± 8.4	3.05 ± 4.4
3 timepoints	36	2.20 ± 1.8	2.28 ± 1.9	2.28 ± 1.9	1.91 ± 2.3	1.43 ± 1.3*
4 timepoints	36	2.03 ± 1.7	2.22 ± 1.8	2.21 ± 1.8	1.12 ± 1.5**	1.13 ± 1.0
5 timepoints	29	2.13 ± 1.7	2.33 ± 1.9	2.32 ± 1.8	0.77 ± 1.3**	1.29 ± 1.1
6 timepoints	22	2.11 ± 1.7	2.34 ± 2.0	2.33 ± 2.0	0.62 ± 0.9**	1.21 ± 1.0
7 timepoints	15	1.79 ± 1.5	2.03 ± 1.7	2.01 ± 1.7	0.50 ± 0.6**	0.99 ± 0.8
8 timepoints	11	2.10 ± 1.5	2.36 ± 1.8	2.34 ± 1.7	0.33 ± 0.5**	1.14 ± 0.8
9 timepoints	6	1.82 ± 1.0	2.16 ± 1.2	2.13 ± 1.2	0.23 ± 0.2**	0.93 ± 0.5
10+ timepoints	7	1.50 ± 0.6	1.78 ± 1.0	1.76 ± 0.9	0.05 ± 0.1**	0.75 ± 0.3

Benjamini-Hochberg method was used to adjust *p*-values to account for multiplicity of statistical tests.

†Two-parameter least squares fitting with 3 or more timepoints.

*Statistically significantly different from error in using 1. MIC latest timepoint to calculate *r* with $p < 0.05$.

**Statistically significantly different from error in using 1. MIC latest timepoint to calculate *r* with $p < 0.01$.

***Statistically significantly different from error in using 1. MIC latest timepoint to calculate *r* with $p < 0.001$.

## Discussion

When an ADPKD patient has multiple abdominal CT or MRI scans available for measuring htTKV, it has not been clear which scan should be used to calculate the Mayo Imaging Classification (MIC) to most accurately predict the future htTKV growth rate. Our data from 36 ADPKD subjects followed by imaging for at least 8 years (11 years on average) to establish ground truth htTKV growth rates, show that obtaining MIC from the most recent scan is more accurate than MIC from early scans and there is no benefit from averaging MIC across all the scans. HtTKV growth rate derived from the index scan aligned nearly perfectly with ground truth htTKV growth rates in 20% of ADKPD patients, particularly in females and with *PKD1* mutations. However, MIC predicted htTKV growth rate was substantially misaligned compared to ground truth in the 22 of 36 (60%) ADPKD patients indicating there is opportunity for improvement especially in males and subjects with *PKD2* mutations. When multiple imaging timepoints were available, superior htTKV growth prediction accuracy was achieved using a 2-parameter least squares fit preferably on at least 3 imaging timepoints or imaging follow-up spanning 5 or more years.

We were initially surprised that the MIC from the latest scan was more accurate than averaging MICs from all available scans. It seemed to be intuitive that methods incorporating data from multiple scans would predict htTKV growth rate more accurately. Perhaps the assumption of a fixed htTKV at birth being 150 mL/m is not physiological as noticed by Breysem et al.^21^ creates prediction error in some subjects that cannot be corrected by averaging. We found that the MIC was less accurate in males compared to females which might be explained by increased prevalence of hypertension and possibly males are less well hydrated. In the group of subjects with near perfect alignment with MIC, 43% had normal blood pressure and were all female. For the entire cohort, the majority of patients (71%) with normal blood pressure were female. Greater MIC error in *PKD2* patients may reflect the limited number of *PKD2* mutation patients incorporated into the development of the MIC algorithm, where only 16%, 14%, and 14% of subjects had *PKD2* mutation compared to 77%, 80%, and 78% with *PKD1* mutations included in development, internal validation, and external validation respectively^6^. This raises the possibility that MIC could be separately optimized for *PKD1* vs. *PKD2* mutations. In the meantime, the 2-parameter fit is recommended for estimating TKV growth rate when 3 or more imaging timepoints are available.

The 2-parameter least squares fitting addresses the error of assuming htTKV being 150 mL/m at birth by allowing the least squares fitting to find a more optimum value. This did not work as well as MIC when only two scans were available because that is not sufficient data for optimal fitting. With 3 or more timepoints, 2-parameter least squares fitting overcomes the problem of insufficient data and estimates htTKV trajectory more accurately than MIC particularly in males and in subjects with *PKD2* mutations. Our data show that with 3 timepoints available, taking the average of MIC and the 2-parameter fit outperforms either one alone. Once 4 timepoints are available, the superior accuracy of the 2-parameter fit is so substantial that there is no longer any benefit from averaging together with MIC.

Although having scans at 4 timepoints is challenging, either CT or MRI can be used. In our cohort of 228 active ADPKD patients, 139 (60%) had 4 or more scans and more than 5 years imaging follow-up. Two subjects included in this cohort already had MRI or CT scan(s) at the time of presentation to our institution. This 2-parameter fitting is also more sensitive to TKV measurement errors which we minimized by measuring TKV rigorously, segmenting organs using deep learning on every MRI pulse sequence available so that up to five TKV measurements could be averaged at each timepoint. This process helps to eliminate pulse sequence bias and operator bias^11^. Moreover, for subjects predicted to have less rapid progression who are not treated with tolvaptan, cross-sectional imaging studies may accumulate over multiple years of follow-up. At presentation with only 1 imaging study available, MIC is the only choice for estimating annual TKV growth. But after years of follow-up with more scans available, fitting htTKVs measured from every scan using the 2-parameter fit allows improved risk assessment relative to using MIC on the most recent scan.

Limitations of this study include the small number of subjects with 8 years of imaging follow-up available that may limit generalizability and the retrospective nature of data collection which can cause selection and observation biases. The requirement of a minimum of 8 years follow-up imaging may have led to a bias toward having more *PKD2* patients since some *PKD1* patients reached endpoints excluding their participation before 8 years had elapsed such as end-stage kidney disease, cyst aspirations, and tolvaptan therapy. The variable number of scans and duration between scans may also introduce bias. However, our observations are consistent with previous studies showing the influence of water intake^22^, blood pressure control^23–25^, and genotype effects^2^. We plan to study htTKV progression on a larger cohort as well as connecting this more accurate approach to measuring htTKV annual growth rate with trends of eGFR decline.

In conclusion, MIC provided ideal prediction of future htTKV growth in only 20% of ADPKD subjects with substantial misalignment from ground truth in 60%. There is better prediction accuracy applying 2-parameter fitting when 3 or more scans are available.

## Methods

### Study subjects

This HIPAA compliant study retrospectively reviewing existing patient data was approved by the Weill Cornell Medicine Institutional Review Board which waived the requirement for written informed consent. All methods were performed in accordance with the Declaration of Helsinki and relevant guidelines and regulations. All subjects were diagnosed with ADPKD based upon Pei criteria^26,27^. The picture archival computer system (PACS) and patient records were reviewed to identify the number of CT and MRI studies for each ADPKD patient. Inclusion criteria included at least 4 abdominal imaging studies (MRI or CT) and imaging follow-up of 8 or more years. Exclusion criteria were atypical radiographic features of ADPKD (e.g., asymmetric cyst distribution), solitary kidney, tolvaptan therapy, kidney replacement therapy (i.e., dialysis, kidney transplantation), history of cyst aspiration/rupture or incomplete data in medical records. Years of imaging follow-up for the patient cohort in this study correlate slightly with the number of images (Supplemental Fig. S2), where on average, an abdominal imaging study was performed every 2 years.

Patient date of birth, biological sex, height, serum creatine, history of tolvaptan therapy, dialysis, transplantation, interventional procedures were obtained from the electronic medical record. Estimated glomerular filtration rate (eGFR) was calculated by CKD-EPI equation 2021^28^.

The presentation of the study fulfilled the STROBE checklist^29^.

### *PKD1/2* gene testing for pathogenic mutations

Puregene®Blood Core Kit (Qiagen, Germantown, MD) was used to extract genomic DNA from peripheral blood lymphocytes. Long-range PCR next-generation sequencing according to Tan et al. with minor modifications^30^. LR-PCR included amplification of the entire coding region, exon-intron boundaries, and boundary 5’ and 3’ untranslated regions of *PKD1* (NM_001009944.3) and *PKD2* (NM_000297.4) in a total of ten distinct PCR reactions (five reactions per gene). LR-PCR used primers that hybridize either to the single-copy region of *PKD1* or in regions distinct from homologues and pseudogenes. The Illumina MiSeq system (Illumina, San Diego, CA) then sequenced the constructed libraries using paired-end 250-bp cycle parameters according to the manufacturer’s instructions. In-house analysis software used Genome Analysis Toolkit (GATK, version 3.5) Best Practices Workflow to generate BAM files mapped to the human reference genome (GRCh37) via the Burrows-Wheeler Aligner (BWA) with further processing in the Picard pipeline. Computational prediction tools, including PolyPhen2, SIFT, Align-GVGD, CADD, SSPNN, Human Splicing Finder, and SpliceAI supplied analysis of variant pathogenicity. Personnel then cross-referenced these values with information in ClinVar, Mayo PKD database, the Rogosin Institute ADPKD Database, gnomAD, and literature on specific variants while following interpretation guidelines by ACMG and AMP to classify mutations^31^.

### htTKV measurements from CT/MR abdomen images

TKV was measured using kidney contours corrected by an expert observer (Z.H.) based on inferences from a deep learning algorithm developed by He et al., where left and right kidneys were segmented on all available pulse sequences if fully included in the field of view of the image (i.e. segmentation mask does not intersect with the boundary of the image) for each MRI and CT study^11^. For each MRI, the final TKV was computed by averaging the TKVs measured from axial or coronal acquisitions of T1, T2 or balanced steady-state free precession. Quality control methods including checking standard deviations of kidney volumes across multiple sequences were used to ensure accurate volume measurements. HtTKV was calculated by dividing TKV at each time point by the patient height (in meters).

### Calculating annual htTKV growth rate, *r*

Equation (1) was used for modeling kidney volume growth in ADPKD^19^ given the height-adjusted total kidney volume at birth is $\mathrm {htTKV_0}$:

1 $$\begin{aligned} \textrm{htTKV}(t)&= \mathrm {htTKV_0} \cdot (1+r)^t. \end{aligned}$$

Four methods to calculate *r* were identified and investigated:

- Method 1: Mayo Imaging Classification (MIC) formula, assumes htTKV_0_ (at birth) $= 150$ mL/m^6^;
- Method 2: same as Method 1 but averaging over all imaging studies;
- Method 3: 1-parameter least squares fit including MIC assumption that htTKV_0_ (at birth) $= 150$ mL/m;
- Method 4: 2-parameter least squares fit without any assumptions on the htTKV at birth.

**Figure 2:**
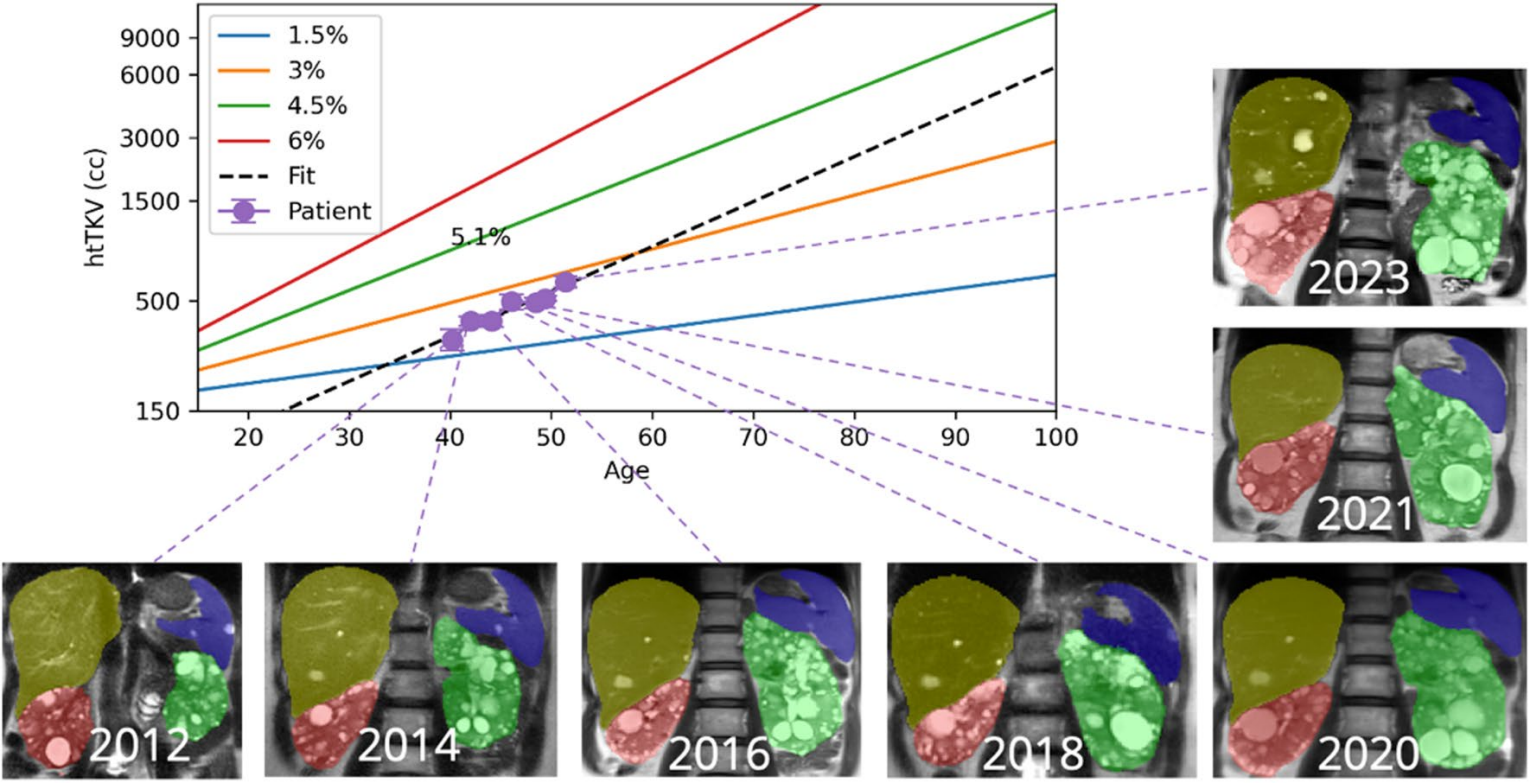
Ground truth htTKV growth trajectory derived from 11+ years imaging follow-up showing 5.1% growth per year, corresponding to Mayo Imaging Classification 1D. This was substantially different from the 1B Mayo Imaging Classification calculated using any of the individual scans. Organs segmented were left kidney (green), right kidney (red), liver (yellow) and spleen (blue).

**Figure 3:**
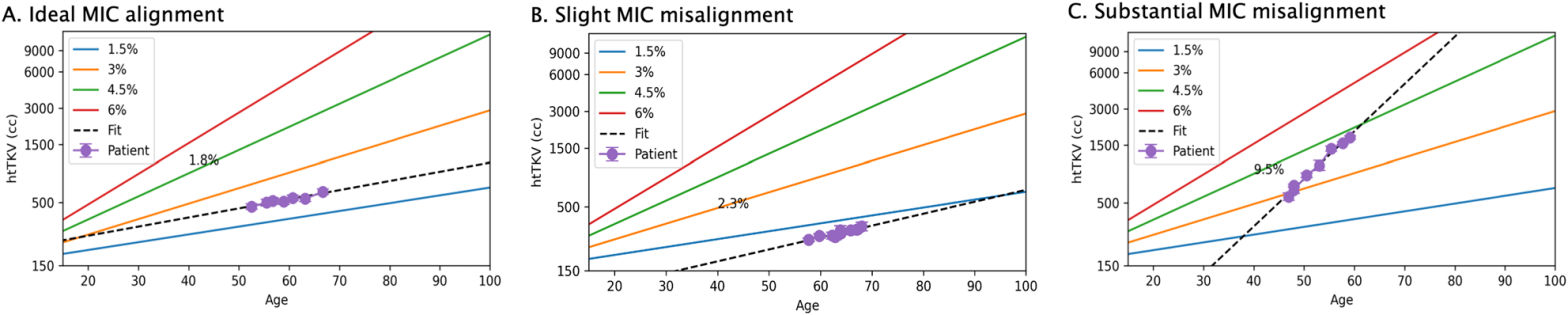
Examples of (**A**) Ideal alignment: subject has a true annual height adjusted total kidney volume (htTKV) growth rate (*r*) of 1.8% which lies between the bounds defined by Mayo Imaging Class 1B ($1.5\% \le r < 3\%$); (**B**) slight misalignment: subject has a true annual htTKV growth rate of 2.3% yet is classified as Mayo Imaging Class 1A ($r < 1.5\%$), yet all points belong to Mayo Imaging Class 1A; (**C**) substantial misalignment: subject has a true annual htTKV growth rate of 9.5% and the Mayo Imaging Class ranges from 1B to 1C/1D.

The ground truth annual htTKV growth rate was calculated by fitting all available htTKVs to Eq. (1) as shown in Fig. 2. The accuracy of the average of htTKV increase rate calculated using Methods 1 and 4 was also evaluated. More detailed derivation of Methods 1-4 can be found in Supplemental Methods.

### Prediction accuracy assessment

Patients were divided into three groups (ideal alignment, slight misalignment and substantial misalignment) based on how well their ground truth htTKV growth trajectories fit with the MIC calculation for the first available scan. Ideal alignment was defined as a mean annual growth rate predicted by the MIC calculation being within 0.75% per year from ground truth (thickness of 1 MIC class band). MIC slight misalignment varies $\ge$ 0.75% but less than 1.5% from ground truth (worse than ideal alignment but still within 2 class bands). Substantial misalignment varied $\ge$ 1.5% from ground truth and in these patients the MIC was commonly calculated to be different classes at different timepoints. Example subjects for each of these groups are illustrated in Fig. 3.

To assess the relative accuracy of the 4 methods of calculating htTKV, we simulated the clinical situation of the subjects presenting with 2 scans, then with 3 scans then with 4 scans and so on. Since the MIC averaging (Method 2) and the 1- and 2-parameter least squares fitting (Methods 2 and 3) required at least two scans, it was not possible to compare the methods for the index scan. So for each of the 36 patients we could compare performance of the 4 methods on all but the first scan. This yielded a total of 198 assessments. As a first comparison these 198 assessments were averaged for each method. Then we grouped them by genotype, sex, years of follow-up and number of timepoints for further subgroup analyses.

### Statistics

The significance of error reduction in htTKV growth rate measured by MIC and 2-parameter least squares fitting under various conditions was determined via paired T-test. The *p*-values of difference in the age at index for ideal MIC alignment versus substantial MIC misalignment were calculated via Mann-Whitney test, and the *p*-values of male to female ratio in subjects with ideal MIC alignment and subjects with substantial MIC misalignment were calculated via Fisher’s exact test. The significance in the difference between rate of having *PKD2* mutation and substantially MIC misalignment versus rate of having *PKD2* mutation and ideal MIC misalignment were determined was calculated using the exact Poisson method. A sensitivity test on the effect of genotype was also performed for the subgroup of patients with 10+ years of imaging follow-up ($n = 25$), and similar result was obtained that patients with *PKD2* mutations are more likely to be substantially misaligned from MIC ($p = 0.03$). Similarly, the prevalence of normal blood pressure was assessed using the exact Poisson method. To account for multiplicity, Benjamini-Hochberg method was used to correct *p*-values for each group of assessments.

### Supplementary Information

Supplementary Information.

## Data Availability

The longitudinal imaging data on the 36 patients can be shared via a Weill Cornell data sharing agreement upon request, please contact the corresponding author.
