# Supplementary material for: Improved predictions of total kidney volume growth rate in ADPKD using two-parameter least squares fitting
Source: Sci Rep. 2024 Jun 14;14:13794. doi: 10.1038/s41598-024-62776-8 (PMC11178802; doi:10.1038/s41598-024-62776-8)
Supplement: Supplementary file 1 — Supplementary Information. [file 41598_2024_62776_MOESM1_ESM.pdf]

Improved Predictions of Total Kidney Volume Growth Rate in ADPKD using Two-parameter Least Squares Fitting

Supplementary Materials

Hu *et al.*

April 23, 2024

S1 Supplemental Figures

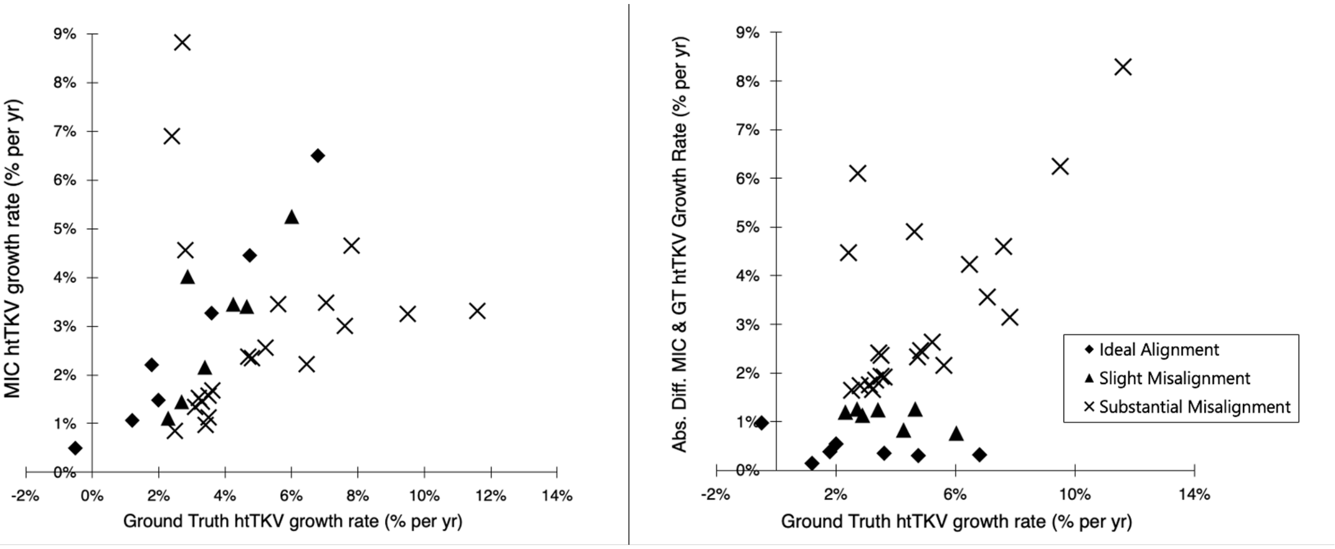

Figure S1: Height adjusted total kidney volume (htTKV) growth rate measured by Mayo Imaging Classification on first scan available versus Ground Truth. Subjects were categorized as ideal alignment (solid diamond), slight misalignment (solid triangle), substantial misalignment (X). (Left) htTKV growth rate calculated by Mayo Imaging Classification from the initial, index scan versus ground truth htTKV growth rate. (Right) Absolute difference between Mayo Imaging Classification htTKV growth rate from the index scan versus ground truth htTKV growth rate.

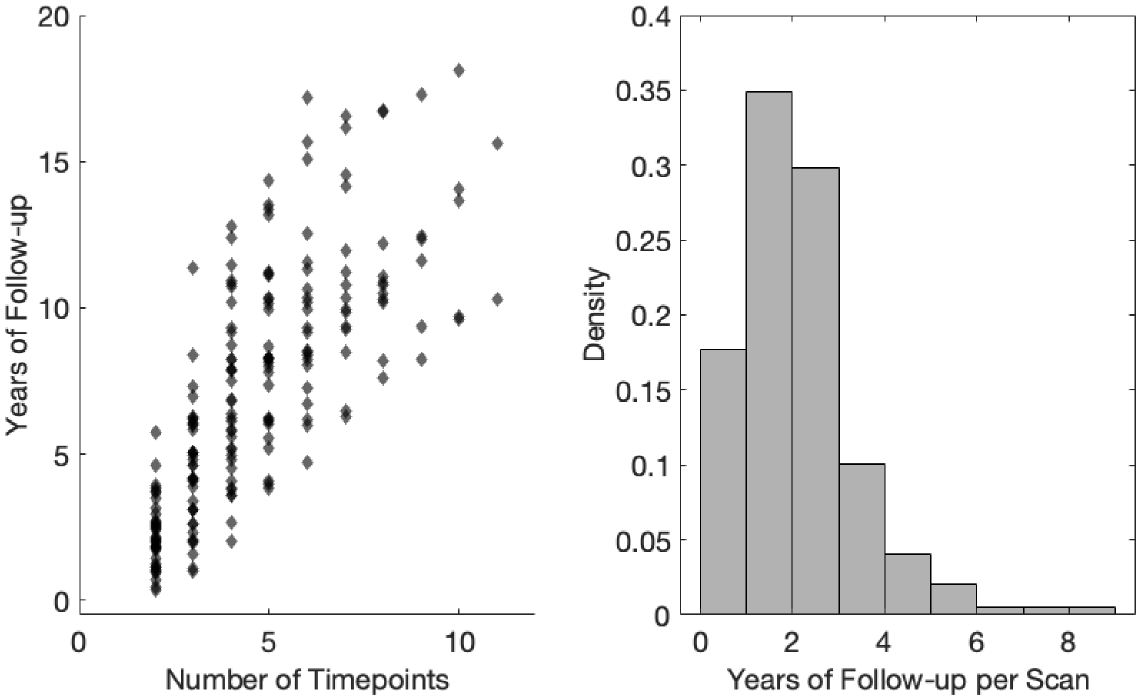

Figure S2: Correlation between Years of Follow-up and Number of Timepoints for $n = 36$ ADPKD Patient Cohort. (Left) Years of follow-up versus number of timepoints. (Right) Distribution of years of follow-up per scan. On average, patients included in this cohort obtained a new imaging follow-up every two years.

S2 Supplemental Methods

Methods to Calculate Annual htTKV Growth Rate

Kidney volume growth in ADPKD can be modeled by Equation S1 given the height-adjusted total kidney volume at birth is htTKV_0,

$$\mathrm{htTKV}(t) = \mathrm{htTKV}_0 \cdot (1+r)^t. \tag{S1}$$

Four methods to calculate r were identified and investigated:

- Method 1: Mayo Imaging Classification (Irazabel et al.), assumes $\mathrm{htTKV}_0 = 150\,\mathrm{mL/m}$, infers htTKV increase rate (r_1) to be:

$$r_1 = \exp\left(\frac{1}{t_i}[\ln(\mathrm{htTKV}_i) - \ln(150\,\mathrm{mL/m})]\right) - 1;$$

- Method 2: average of MIC calculated from n available imaging studies. The htTKV increase by rate r_2:

$$r_2 = \frac{1}{n}\sum_{i=1}^{n} \exp\left(\frac{1}{t_i}[\ln(\mathrm{htTKV}_i) - \ln(150\,\mathrm{mL/m})]\right) - 1;$$

- Method 3: 1-parameter least squares fitting which minimizes sum of square residuals under assumption $\mathrm{htTKV}_0 = 150\,\mathrm{mL}$. Equation S1 can be linearized and the distance from measurements to fit can be minimized:

$$\ln(\mathrm{htTKV}(t)) = \ln(150\,\mathrm{mL/m}) + t \cdot \ln(1+r).$$

  The R^2 of the fit can then be written as $R^2(r)$ and reaches its maximum when $dR^2(r)/dr = 0$:

$$R^2(r) = \sum_{i=1}^{n}[\ln(\mathrm{htTKV}_i) - (\ln(\mathrm{htTKV}_0) + t_i \ln(1+r))]^2,$$

$$\frac{d(R^2)}{dr} = \sum_{i=1}^{n} \frac{2t_i}{1+r} \cdot (\ln(150\,\mathrm{cc}) + t_i \ln(1+r) - \ln(\mathrm{htTKV}_i)).$$

  Therefore, rate of htTKV increase can be estimated as r_3:

$$r_3 = \exp\left(\frac{\sum_{i=1}^{n}[\ln(\mathrm{htTKV}_i) - \ln(150\,\mathrm{mL/m})]}{\sum_{i=1}^{n} t_i}\right) - 1.$$

- Method 4: 2-parameter least squares fitting which minimizes sum of square residuals without any assumption. Similarly, if htTKV_0 is no longer restricted and given that $y_i = \ln(\mathrm{htTKV}_i)$, R^2 is maximized when

$$r_4 = \exp\left(\frac{\sum_{i=1}^{n} t_i y_i - n\bar{t}\bar{y}}{\sum_{i=1}^{n} t_i^2 - n(\bar{t})^2}\right) - 1,\ \mathrm{htTKV}_0 = \exp\left(\frac{\bar{y}\sum_{i=1}^{n} t_i^2 - \bar{t}\sum_{i=1}^{n} t_i y_i}{\sum_{i=1}^{n} t_i^2 - n(\bar{t})^2}\right).$$
